# Editorial: Neural inspired computational intelligence and its applications

**DOI:** 10.3389/fnins.2024.1515039

**Published:** 2024-12-12

**Authors:** Ying Tan, Yuhui Shi, Yan Pei

**Affiliations:** ^1^Peking University, Beijing, China; ^2^Southern University of Science and Technology, Shenzhen, Guangdong, China; ^3^University of Aizu, Aizuwakamatsu, Japan

**Keywords:** neural network, bio-inspiration design, intelligence theory, optimization algorithm, computational intelligence

Computational intelligence (CI) should be the hottest research field in modern artificial intelligence, which has been experienced remarkable advancements through the integration of bio-inspired neural network models. These models, drawing inspiration from biological systems, have shown great promise in addressing complex computational challenges and enhancing our understanding of neural processes. This Research Topic, “*Neural inspired computational intelligence and its applications*,” explores the latest technologies and methodologies in neural network-based computational intelligence and their advanced applications, including neural network architectures, face perception, synaptic plasticity, swarm intelligence, and the theoretical foundations of intelligence science. Actually, CI, as a vital branch of artificial intelligence, tackles complex problems by emulating human intelligence and natural phenomena, with wide-ranging applications in areas such as healthcare and finance, including disease diagnosis and stock market prediction. As computational power and data volumes continue to grow, CI will increasingly integrate with big data, the Internet of Things (IoT), and cloud computing, driving advancements in smart cities, intelligent transportation, and smart healthcare. Looking ahead, CI holds vast potential for application and development, poised to play a crucial role in technological progress and societal advancement. The following section highlights five of the most cutting-edge studies in this field, covering several important research aspects of computational intelligence.

The first article, entitled “*Bio-inspired circular latent spaces to estimate objects' rotations*,” by Plebe and Da Lio, presents a novel neural network model designed to estimate the rotation angle of unknown objects from RGB images. Inspired by the rotational representation in the ellipsoid body of Drosophila, the proposed model embeds the understanding of rotational transformations into its architecture. The network's latent space is structured in a circular manner to effectively capture the cyclic nature of rotation. The rotation operator acts as a shift in the circular latent space's units, establishing a direct correspondence between shifts in the latent space and angular rotations of the object in the world space. Remarkably, the model accurately estimates the difference in rotation between two views of an object, even for categories of objects it has never seen before. Furthermore, it outperforms three state-of-the-art convolutional networks commonly used as the backbone for vision-based models in robotics. This innovative approach not only enhances our understanding of rotational transformations but also sets a new benchmark for performance in this domain.

The second article, entitled “*Understanding of facial features in face perception: insights from deep convolutional neural networks*,” by Zhang et al., delves into the mechanisms of face perception through the lens of deep convolutional neural networks (CNNs). By analyzing how CNNs process facial features, the study provides valuable insights into the relative importance of different facial features in identifying individuals. Previous studies in humans have demonstrated the crucial role of eyebrows in face recognition, potentially even surpassing the importance of the eyes. This research not only advances our understanding of face perception but also has practical implications for improving facial recognition technologies.

In the third article, entitled “*Context association in pyramidal neurons through local synaptic plasticity in apical dendrites*,” by Baronig and Legenstein, the focus shifts to the biological underpinnings of neural computation. The study investigates how local synaptic plasticity in the apical dendrites of pyramidal neurons contributes to context association. By modeling these processes, the research offers a deeper understanding of how neurons integrate contextual information. The resulting plasticity rule utilizes information available either locally at the synapse or through global Ca^2+^ events, both observed experimentally in layer 5 pyramidal cells. Computer simulations show that this plasticity rule enables pyramidal cells to associate top-down contextual input patterns with high somatic activity, perform context-dependent tasks, and enable continual learning by allocating new dendritic branches to novel contexts.

The fourth article, entitled “*An efficient swarm intelligence approach to the optimization on high-dimensional solutions with cross-dimensional constraints, with applications in supply chain management*,” by Liu et al., explores the application of bio-inspired swarm intelligence to complex optimization problems. The proposed approach addresses high-dimensional solutions with cross-dimensional constraints, demonstrating its effectiveness in supply chain management scenarios. This research highlights the versatility and practical utility of bio-inspired computational methods in solving real-world problems, particularly in the context of e-commerce and supply chain optimization.

Finally, the fifth article, entitled “*Eight challenges in developing theory of intelligence*,” by Huang, identifies and discusses key challenges in developing a comprehensive theory of intelligence. The article emphasizes the importance of bottom-up mechanistic modeling in understanding both natural and artificial intelligence. The identified challenges include representation learning, generalization, adversarial robustness, continual learning, causal learning, internal model of the brain, next-token prediction, and the mechanics of subjective experience. Addressing these challenges provides a roadmap for future research in both artificial and biological intelligence, emphasizing the need for interdisciplinary collaboration and innovative approaches.

In summary, these articles showcase the cutting-edge advancements in neural-inspired computational intelligence and its applications. The insights and innovations presented not only summarize the current state of the field but also highlight the broad application prospects and future directions. From enhancing robotic vision systems to optimizing complex supply chain networks, and from understanding the intricacies of face perception to developing a comprehensive theory of intelligence, the potential applications are vast and varied. We hope that the insights and innovations presented in this Research Topic will inspire further research and development in the exciting and rapidly evolving fields, paving the way for new breakthroughs and practical solutions in computational intelligence.

